# The Dissector, or Practical and Surgical Anatomy

**Published:** 1843-11-25

**Authors:** 


*The Dissector, or Practical and Surgical Anatomy.* By Erasmus Wilson, Author of a "System of Human Anatomy," etc. *With one hundred and six Illustrations. Modified and Rearranged.* By Paul B. Goddard, M. D., Demonstrator of Anatomy in the University of Pennsylvania. Philadelphia: Lea and Blanchard. 1844. 12mo. pp. 444.

This is a well printed large duodecimo volume, copiously illustrated, with the descriptions clearly and tersely written. The work "has been carefully modified in such a manner that the student, by following the order of parts as they are laid down, will obtain the utmost advantage which can be derived from a single subject."—(Preface to the American edition). From careful inspection we should say that the young dissector could not have a better guide, and its selection from other English works of the same class by Dr. Goddard, is strong evidence in favor of its excellence.

